# Blood Coagulation Factor X Exerts Differential Effects on Adenovirus Entry into Human Lymphocytes

**DOI:** 10.3390/v10010020

**Published:** 2018-01-03

**Authors:** James S. Findlay, Graham P. Cook, G. Eric Blair

**Affiliations:** 1 School of Molecular and Cellular Biology, University of Leeds, Leeds LS2 9JT, UK; jsfindlay@dstl.gov.uk; 2 Leeds Institute of Cancer and Pathology, University of Leeds, St. James’s University Hospital, Leeds LS9 7TF, UK; g.p.cook@leeds.ac.uk

**Keywords:** adenovirus, factor X, lymphoid cells, blood lymphocytes, virus entry

## Abstract

It has been proposed that blood coagulation factors, principally factor X (FX), enhance the uptake of human adenovirus type 5 (Ad5) into cultured epithelial cells by bridging the viral hexon capsid protein and cell-surface heparan sulphate proteoglycans (HSPGs). We studied the effects of FX on Ad transduction of lymphoid cell lines (NK92MI, a natural killer cell line; Daudi, a B-cell line and Jurkat, a T-cell line) as well as primary peripheral blood lymphocytes (PBL) and HeLa epithelial cells using either replication-deficient Ad5, or a derivative in which the Ad5 fiber was replaced with that of another Ad type, Ad35, termed Ad5F35. PBL and NK92MI were resistant to Ad5 transduction. Transduction of Jurkat and Daudi cells by Ad5 was reduced by FX but without discernible effects on cell-surface Ad5 binding. FX reduced virus binding and transduction of all lymphoid cell lines by Ad5F35, as well as transduction of the T- and Natural Killer (NK)-cell populations of PBL. Flow cytometry analysis showed that all lymphoid cell lines were negative for HSPG components, in contrast to HeLa cells. FX reduced transduction of an HSPG-negative mutant Chinese hamster ovary cell line (CHOpgsA745) by Ad5 and Ad5F35, with Ad5F35 binding also being reduced by FX. These results point to fiber-dependent differences (Ad5 versus Ad35 fiber) in Ad binding to and transduction of human lymphoid and epithelial cells in the presence of FX.

## 1. Introduction

Extensive studies in cultured cells in vitro have shown that human adenoviruses (Ads) enter human cells by a two-step process: a primary interaction between the Ad fiber and cell-surface attachment molecules followed by an interaction between the penton base and cell-surface integrins α_v_β_3_ and α_v_β_5_. The three major cell-surface attachment molecules used by Ads in vitro are the Coxsackie and adenovirus receptor, CAR, (utilized by most Ads including type 5, Ad5), CD46 (a complement inhibitor protein utilized by certain types such as Ad35) and Desmoglein 2 (utilized by types such as Ad3) [1,2,3]. In contrast, intravenous delivery of Ad5 in rodents and nonhuman primates resulted in attachment molecule-independent accumulation of virus in the liver [4,5,6]. Further studies showed that blood coagulation factor X (FX) mediated liver tropism in vivo [7,8]. FX binds to hypervariable regions of the major capsid protein, the hexon, and has been proposed to act as a bridge between heparan sulphate proteoglycans (HSPGs) on target cells and hexon trimers in the virus capsid [9,10,11,12,13]. However, mice in which Ext1 (an enzyme required for HS biosynthesis) was deleted showed no significant reduction in liver transduction in vivo by Ad5, thus questioning a role for HSPGs in Ad entry into cells in vivo [14]. A hybrid Ad, in which the Ad5 fiber was replaced with that of Ad35 (Ad5F35), exhibited reduced liver and increased lung tropism in the presence of FX [15].

Investigations of Ad interactions with cells and proteins of the blood are important for their application as safe and efficient viral gene therapy vectors that can be delivered intravenously. Such studies should lead to the design of modified viruses that combine the required tissue tropism and optimal levels of transduction. Hybrid viruses can also be useful tools in dissecting differences in the activities of specific proteins of different Ad types in the context of whole virus genomes.

Previous studies have shown that human blood lymphocytes and certain lymphoid cell lines are relatively refractory to transduction by Ad5 but are readily transduced by Ad5F35, although the influence of FX in this cell system has not been examined [16,17,18,19,20,21].

In this study, we have investigated the interactions between Ads (Ad5 and Ad5F35), blood coagulation factors (principally FX) and lymphoid cells. In contrast to previous studies on human epithelial cells, we find that FX treatment of lymphoid cell lines and PBL reduces transduction by Ad5F35, an effect that is accompanied by reduced virus binding to cells. The lymphoid cell lines found here to express very low levels of cell-surface HSPG and an HSPG-negative Chinese hamster ovary (CHO) cell line (in which HSPG biosynthesis was genetically ablated) also showed reduced levels of transduction and virus binding by Ad5F35 when treated with FX. The lack of enhancement of Ad binding to lymphoid cells in the presence of FX can be explained by their greatly reduced level of HSPG. However the mechanism of FX-mediated inhibition of Ad5F35 binding and cell transduction requires further investigation. Thus there are fiber-dependent and cell-specific effects of FX on Ad entry. Our results suggest that plasma FX may exert a protective effect on lymphoid cells against Ad entry.

## 2. Materials and Methods

### 2.1. Materials

FX and FXII were purchased from Haematologic Technologies (Essex Junction, VT, USA). The Fluorescein isothiocyanate (FITC)-labelled antihuman HSPG antibody termed 10E4 (clone 8.S.087) was purchased from US Biologicals (Salem, MA, USA), the antihuman CAR antibody (clone E1-1) was purchased from Abcam (Cambridge, UK) and the FITC-labelled antihuman CD46 (clone E4.1), PE-labelled antihuman CD19 (clone HIB19), APC-labelled antihuman CD56 (clone B159) and APC-Cy7 labelled antihuman CD3 (clone SK7) antibodies were purchased from BD Bioscience (Oxford, UK).

### 2.2. Cell Lines

HeLa cells (obtained from the European Collection of Authenticated Cell Cultures (ECACC), Salisbury, UK) and CHO cells were cultured in Dulbecco’s Minimal Eagle Medium (DMEM; Sigma-Aldrich, Dorset, UK) supplemented with 10% Foetal Bovine Serum (GE Healthcare, Buckinghamshire, UK), 2 mM l-glutamine, 50 units penicillin/mL and 50 µg streptomycin/mL (Sigma-Aldrich); this supplemented DMEM is hereafter referred to as “complete DMEM”. CHOpgsA745 cells (a kind gift from Prof. David J Evans, University of Glasgow, Glasgow, UK) were cultured in Ham’s F12 medium (Sigma-Aldrich) supplemented with 10% Foetal Bovine Serum (GE Healthcare, Little Chalfont, UK), 2 mM l-glutamine, 50 units penicillin/mL and 50 ng streptomycin/mL (Sigma-Aldrich). NK92MI, Daudi and Jurkat cells were cultured in Roswell Park Memorial Institute 1640 Media (RPMI; Sigma-Aldrich) supplemented with 10% Foetal Bovine Serum (GE Healthcare), 2 mM l-glutamine, 50 units penicillin/mL and 50 ng streptomycin/mL (Sigma-Aldrich); supplemented RPMI 1640 is hereafter referred to as “complete RPMI”. Adherent cells were detached using trypsin/EDTA (Sigma-Aldrich). All cells were grown at 37 °C in a humidified atmosphere of 5% CO_2_.

### 2.3. Viruses

Ad5-EGFP (Enhanced Green Fluorescent Protein) is a replication-deficient E1- and E3-deleted adenovirus based on Ad5 in which the E1 region was replaced with a cytomegalovirus (CMV) promoter-driven EGFP transgene and was a kind gift from Prof. Aviva M Tolkovsky, University of Cambridge, UK [22]. Ad5F35-EGFP is identical to Ad5-EGFP virus except that the Ad35 fiber replaced the Ad5 fiber and was kindly provided by Prof. Masatoshi Tagawa (Chiba, Japan) [23]. The viruses were propagated in 911 cells and purified using the freeze-thaw method followed by caesium chloride centrifugation [24]. The number of virus particles (vp) was determined by spectrophotometry, as previously described [25].

### 2.4. Flow Cytometric Detection of Cell-Surface Molecules

Adherent cell lines were detached using Versene (Sigma-Aldrich) followed by addition of complete DMEM, centrifugation at 350× *g* for 5 min and resuspension in Phosphate-buffered saline (PBS) + 1% Bovine Serum Albumin (BSA). Cell lines growing in suspension were centrifuged at 350× *g* for 5 min and resuspended in PBS + 1% BSA. Each sample for flow cytometry comprised 2.5 × 10^5^ cells. Cells were incubated with 1% (final concentration) mouse serum (for CD46) or goat serum (for CAR) for 10 min on ice (to block non-specific immunoglobulin binding sites) followed by addition of PBS. Cells were collected by centrifugation (350× *g*, 5 min) and incubated with FITC-mouse-anti-CD46 or mouse anti-CAR for 30 min on ice, washed twice in PBS with centrifugation (350× *g*, 5 min). Binding of primary CAR antibody was detected by incubation for 30 min on ice with a FITC-labelled goat anti-mouse Ig antibody. Cells were washed twice in PBS with centrifugation (350× *g*, 5 min), analyzed by flow cytometry on a FACSCalibur, (BD Bioscience, Wokingham, UK) and the data analyzed using FlowJo software (Tree Star, Ashland, OR, USA).

### 2.5. Transduction of Lymphoid Cell Lines

Cells (2.5 × 10^5^) were centrifuged at 350× *g* for 5 min and washed once with PBS. The supernatant was removed, cells were resuspended in serum-free RPMI and exposed to Ad5-EGFP or Ad5F35-EGFP along with FX or FXII (1 unit/mL final concentration). Cells were incubated for one hour at 37 °C in a humidified atmosphere with 5% CO_2_, 1 mL of complete RPMI 1640 medium was added and incubated at 37 °C for a further 24 h in a humidified atmosphere with 5% CO_2_. The cells were collected by centrifugation, washed in PBS with centrifugation (350× *g*, 5 min) and resuspended in PBS. Cells were analyzed by flow cytometry as described above.

### 2.6. Transduction of Adherent Cell Lines

Cells (5 × 10^4^) were plated in 6 well plates and incubated for 24 h at 37 °C in a humidified atmosphere with 5% CO_2_. The cells were washed with PBS followed by addition of Ad5-EGFP or Ad5F35-EGFP in serum-free DMEM (for CHO and HeLa cells) or Ham’s F12 (for CHOpgsA745 cells) with appropriate supplements: FX or FXII (1 unit/mL). The cells were incubated for one hour at 37 °C in a humidified atmosphere with 5% CO_2_, complete DMEM or Ham’s F12 added and incubated for a further 24 h in a humidified atmosphere with 5% CO_2_. The cells were detached by addition of trypsin-EDTA followed by neutralization with “complete” medium, collected by centrifugation, washed by addition of PBS with centrifugation and resuspension in PBS. Cells were analyzed by flow cytometry as described above.

### 2.7. Fluorescent Labelling of Adenoviruses

Virus particles (1.1 × 10^12^) were dialysed in Molecular Weight Cut-Off (MWCO) 7000 dialysis cassettes (ThermoFisher Scientific, Warrington, UK) against 10 mM 4-(2-hydroxyethyl)-1-piperazineethanesulphonic acid (HEPES)-KOH (pH 8.0) to remove glycerol. Alexa Fluor 488 carboxylic acid succinimidyl ester (50 µg, Invitrogen, Carlsbad, CA, USA) was added and incubated for 1 h on a vibrating platform. The reaction was quenched using 1.5 M hydroxylamine hydrochloride and incubated for 30 min on a vibrating platform. Excess fluorophore was removed by overnight dialysis as described above.

### 2.8. Virus: Cell Binding

Adherent cells were detached by incubation with Versene at 37 °C, addition of complete medium and centrifugation (350× *g*, 5 min). The cells were washed by addition of PBS and centrifugation at 350× *g* for 5 min, resuspended in binding buffer (PBS + 0.5% BSA + 1 mM MgCl_2_ + 1 mM CaCl_2_) and incubated with Alexa Fluor 488-labelled viruses for 1 h on ice. The cells were washed twice by addition of binding buffer with centrifugation at 350× *g* for 5 min and analyzed by flow cytometry as described above.

### 2.9. Isolation of Peripheral Blood Mononuclear Cells (PBMC)

Blood samples were collected following the receipt of informed consent and ethical review by the Leeds Teaching Hospitals National Health Service Trust (REC number 10-H1306-7, awarded 7 January 2010). Peripheral venous blood (12 mL) was removed from healthy donors and collected in Vacutainer Blood Collection tubes (BD Bioscience). The blood was diluted with an equal volume of sterile PBS, layered onto 15 mL Lymphoprep (Axis-Shield, Dundee, UK) at room temperature in a 50 mL centrifuge tube and centrifuged at 850× *g* for 20 min at 20 °C without braking. The resulting cloudy layer in the tube was transferred to a 50 mL centrifuge tube, 40 mL PBS was added and centrifuged at 200× *g* for 10 min at 20 °C. The supernatant was carefully removed by inverting the tube and the cells resuspended in 10 mL PBS.

### 2.10. Transduction of PBMC

PBMCs (2.5 × 10^5^) were centrifuged at 350× *g* for 5 min at 4 °C, the supernatant removed and the cells resuspended in either Ad5-EGFP or Ad5F35-EGFP in serum-free RPMI with or without 1 unit FX/mL and incubated for one hour at 37 °C in a humidified atmosphere with 5% CO_2_. Complete RPMI 1640 was added to each sample and incubated for a further 24 h at 37 °C in a humidified atmosphere with 5% CO_2_. The cells were centrifuged at 350× *g* for 5 min at 4 °C, resuspended in 1 mL PBS and centrifuged at 350× *g* for 5 min at 4 °C. The supernatant was removed and Allophycocyanin (APC)-Cy7 anti-CD3, APC anti-CD56 and Phycoerythrin (PE) anti-CD19 were added. Cells were incubated on ice for 30 min and washed twice by addition of 1 mL PBS with centrifugation at 350× *g* for 5 min at 4 °C. The cells were resuspended in 500 µL PBS, 3 µL propidium iodide (PI) solution (Sigma-Aldrich, 1 mg/mL) was added and incubated for 20 min at room temperature. The samples were analyzed by flow cytometry using a FACSAria (BD Bioscience, Wokingham, UK) and the data analyzed using DiVa software (BD Bioscience, Wokingham, UK).

## 3. Results

### 3.1. Adenovirus Transduction of Lymphoid Cell Lines

**Figure 1:**
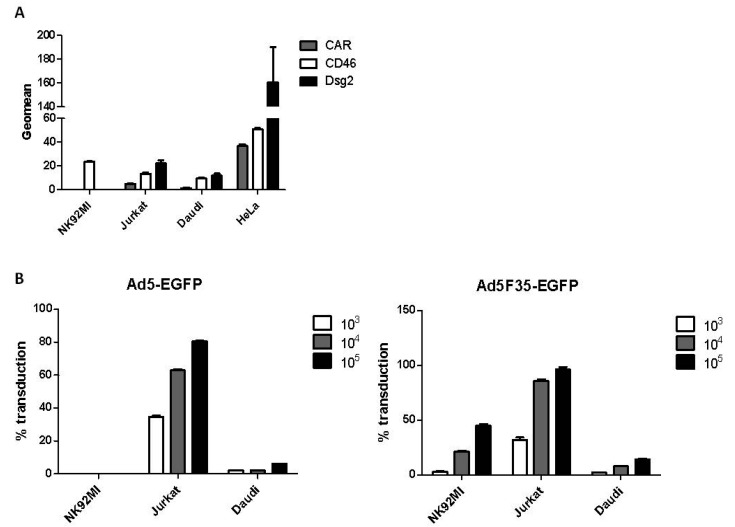
Expression of cell-surface adenovirus attachment molecules and transduction of lymphoid cell lines. (**A**) The expression of the Coxsackie and Adenovirus Receptor (CAR), Desmoglein-2 (Dsg-2) and cluster of differentiation 46 (CD46) was measured on a panel of lymphoid cell lines (NK92MI, Jurkat and Daudi) using flow cytometry. HeLa cells, which support Ad replication, served as a positive control; (**B**) The lymphoid cells were transduced with either Adenovirus 5-Enhanced Green Fluorescent Protein (Ad5-EGFP) or Ad5F35-EGFP at the indicated vp:cell ratios. EGFP expression was measured after 24 h by flow cytometry, the percentage of EGFP-positive cells was determined and expressed as % transduction. In (**A**,**B**), three experimental replicates were performed (*n* = 3) with all samples assayed in triplicate.

Expression of primary cell-surface attachment molecules required for adenovirus entry was examined in lymphoid cell lines that represent the major immune cells of blood: NK92MI (an NK cell line), Jurkat (a T-cell line) and Daudi (a B-cell line). HeLa epithelial cells (which support Ad replication) were used as a positive control (Figure 1A). Cell-surface CD46 was detected on all cell lines, whereas low levels of CAR and Dsg-2 were present on Jurkat and Daudi cells. NK92MI cells were negative for CAR and Dsg2. This suggested that Ads that interact with CD46 (such as Ad35) may be able to enter lymphoid cells. Therefore the lymphoid cell lines were incubated with two replication-deficient viruses, Ad5-EGFP and Ad5F35-EGFP [22,23], at a temperature that permitted transduction (37 °C). Transduction is herein defined as a process that includes virus binding, internalization, trafficking through the cytoplasm to the nuclear pore, transport of the Ad genome to the nucleus and transcription and detection of the product of a virus-encoded expression cassette (EGFP). In both viruses, the essential viral E1A and E1B genes were replaced with the EGFP gene. Ad5F35-EGFP is identical to Ad5-EGFP except that the Ad5 fiber was replaced with that of Ad35. Cells were exposed to each virus at three different vp (virus particle): cell ratios (Figure 1B). Transduction of HeLa cells at the lowest vp:cell ratio (10^3^ vp:cell) with either virus resulted in approximately 100% EGFP-positive cells. Transduction in all cell lines by the CD46-interacting Ad5F35-EGFP occurred in a manner dependent on the vp:cell ratio (Figure 1B). However, only cell lines that expressed CAR showed transduction by Ad5-EGFP, namely Jurkat and (at a much lower level) Daudi cells. CAR-negative NK92MI cells did not exhibit transduction by Ad5-EGFP at any vp:cell ratio. These results indicated that, in the absence of extracellular factors, transduction by Ads was dependent on the presence of cell-surface attachment molecules.

### 3.2. Adenovirus Interactions with Lymphoid and Epithelial Cells in the Presence of Factor X

**Figure 2:**
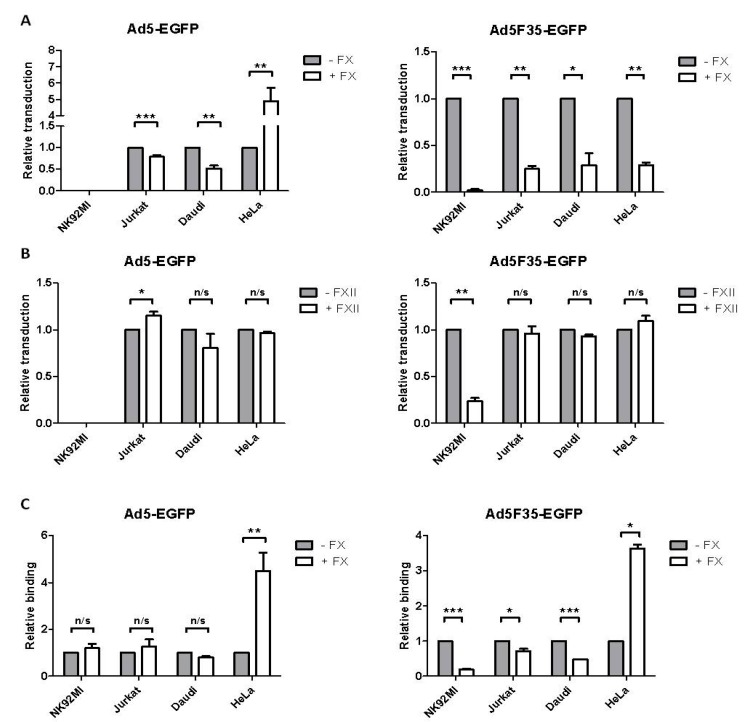
Effect of coagulation factors on adenovirus binding to and transduction of lymphoid and HeLa cells. Lymphoid and HeLa cells were transduced with either Ad5-EGFP or Ad5F35-EGFP (refs [20,21]), at a 10^4^ vp:cell or a 10^3^ vp:cell ratio respectively, in the presence or absence of (**A**) FX or (**B**) FXII. EGFP expression was measured after 24 h by flow cytometry. (**C**) Lymphoid cells were incubated with 10^4^ vp:cell Alexa Fluor 488-labelled Ad5-EGFP or Ad5F35-EGFP on ice for one hour. The cells were washed with PBS and the bound fluorescence measured by flow cytometry. The values given are the geometric mean of either EGFP expression (**A**,**B**) or Alexa Fluor 488 fluorescence (**C**) relative to the geometric mean in the absence of FX. * = *p* < 0.05, ** = *p* < 0.01 and *** = *p* < 0.001 based on a one sample *t* test. Three experimental replicates were performed (*n* = 3) with all samples assayed in triplicate. n/s: not significant.

Host factors such as FX mediate attachment molecule-independent Ad transduction in vivo [6,7,8,9,10,11,12,13]. To investigate possible effects of FX on Ad transduction in lymphoid cells, NK92MI, Jurkat and Daudi cells were incubated with Ad5-EGFP or Ad5F35-EGFP (at a 10^4^ vp:cell ratio, 37 °C) in the presence of a physiological concentration of FX. As a control, HeLa epithelial cells were similarly treated at a 10^3^ vp:cell ratio (Figure 2A). As we previously reported, FX enhanced Ad5-EGFP transduction of HeLa cells [24]; however FX significantly reduced Ad5-EGFP transduction of the Jurkat and Daudi lymphoid cell lines. The presence of FX did not change the refractory nature of NK92MI cells to Ad5-EGFP transduction. FX significantly reduced Ad5F35-EGFP transduction of the lymphoid cell lines as well as HeLa cells. This indicated that FX had not only fiber-specific effects but also cell type-specific effects on Ad transduction of human cells.

To determine if these effects were also shown by any other coagulation factor, the effects of Factor XII (FXII) on Ad transduction were analyzed (Figure 2B). The lymphoid cell lines and HeLa cells were exposed to each Ad in the presence of a physiological concentration of FXII. Interestingly, the presence of FXII resulted in a minor enhancement of Ad5-EGFP transduction of Jurkat, but not Daudi or HeLa cells. Furthermore, FXII significantly reduced Ad5F35-EGFP transduction of NK92MI cells but not Jurkat, Daudi or HeLa cells. Thus a consistent and significant reduction in Ad transduction of the lymphoid cell lines by FXII was not found, in contrast to the generally downregulatory effects of FX on Ad5- and Ad5F35-EGFP-mediated transduction of lymphoid cell lines.

To test if the FX-mediated reduction in transduction involved reduced virus binding at the cell surface, lymphoid and HeLa cells were incubated on ice with Alexa Fluor 488-labelled Ad5-EGFP or Ad5F35-EGFP and bound virus measured by flow cytometry (Figure 2C). FX significantly enhanced Ad5-EGFP binding to HeLa cells but had no effect on binding to the lymphoid cell lines. Ad5F35-EGFP binding to HeLa cells was significantly enhanced in the presence of FX while binding to the lymphoid cell lines was significantly reduced. Therefore, FX also has fiber- and cell type-specific effects on binding of Ads to attachment molecules on the cell surface. Notably, in HeLa cells, FX enhanced both transduction and binding of Ad5-EGFP whereas transduction by Ad5F35-EGFP was reduced by FX, although binding to cells was enhanced. The latter observation might suggest that FX may also modulate intracellular events that are involved in delivering Ad5F35-EGFP to the nucleus of epithelial cells.

### 3.3. Effect of Factor X on Adenovirus Transduction of Primary Blood Lymphoid Cells

**Figure 3:**
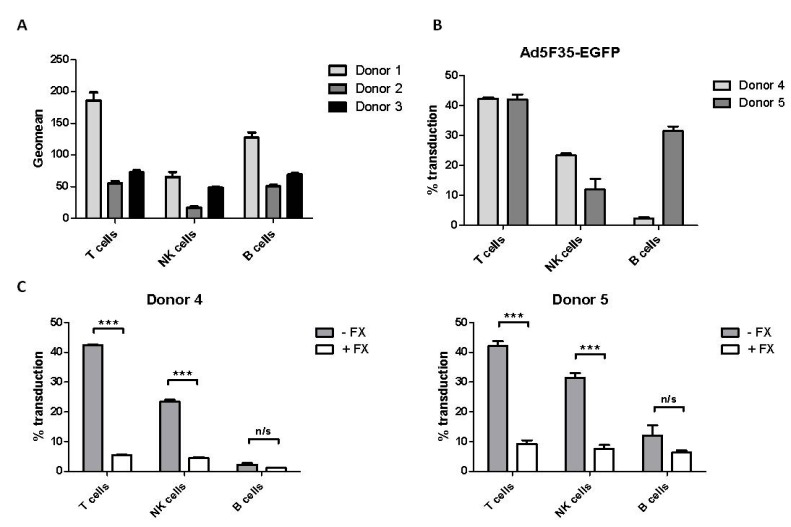
Reduction of Ad5F35-mediated transduction of primary T and Natural Killer (NK) cells by Factor X. (**A**) Peripheral blood lymphocytes (PBL) were isolated from three healthy donors and the expression of CD46 was analyzed on the lymphocyte populations; (**B**) PBL isolated from two healthy donors were incubated with Ad5F35-EGFP at a 10^5^ vp:cell ratio. EGFP expression in the lymphocyte sub-population was measured after 24 h by flow cytometry; (**C**) PBL isolated from the same two healthy donors as in (**B**) and incubated with Ad5F35-EGFP at a 10^5^ vp:cell ratio in the presence or absence of FX. EGFP expression in each lymphocyte population was measured after 24 h by flow cytometry. The lymphocyte populations were identified by flow cytometric detection of cell-surface CD3, CD19 and CD56 using appropriate monoclonal antibodies. B cells were classified as CD19+, NK cells as CD19− CD56+ CD3− and T cells as CD19− CD56− CD3+. *** = *p* < 0.001 based on a two tail student’s *t* test. Two experimental replicates were performed (*n* = 2) with all samples assayed in triplicate. n/s: not significant.

To determine whether the FX-mediated reduction in Ad5-EGFP and Ad5F35-EGFP transduction detected in lymphoid cell lines had physiological relevance, peripheral blood lymphocytes (PBL) were studied. The T-, B- and NK-cell lymphocyte populations were identified and expression of CAR and CD46 was measured by flow cytometry. CAR was not detected in any of these lymphocyte populations. In contrast, CD46 was detected on all lymphocyte populations at levels that varied between donors (Figure 3A). This suggested that incubation of these cells with Ad5F35-EGFP, but not Ad5-EGFP, might result in transduction in the absence of FX. PBL isolated from two different donors (donors 4 and 5) were incubated with either Ad5-EGFP or Ad5F35-EGFP (at a 10^5^ vp:cell ratio), the lymphocyte populations identified and EGFP expression measured. Ad5-EGFP showed no detectable transduction of the lymphocyte populations. Transduction of all lymphocyte populations by Ad5F35-EGFP was detected, particularly in T cells, as previously noted [16], although there was variability between donors (Figure 3B). This indicated that the lymphocyte populations of PBL exhibited transduction by Ad5F35-EGFP in an attachment molecule-dependent manner, consistent with the presence of CD46 on their cell surface. The effect of FX on transduction of PBL by Ad5F35-EGFP was determined (Figure 3C). In both donors 4 and 5, FX treatment resulted in a significant reduction in Ad5F35-EGFP-mediated transduction of T cells and NK cells. While there was some reduction in transduction of B cells in both donors, this did not reach statistical significance. Overall, the results obtained with PBL were similar to those obtained with lymphoid cell lines in terms of a reduction in Ad5F35-EGFP transduction in the presence of FX, giving physiological relevance to the reduction of Ad5F35-EGFP transduction mediated by FX in lymphoid cell lines.

### 3.4. Role of HSPG Expression in FX-Mediated Adenovirus Transduction of Lymphoid Cells

**Figure 4:**
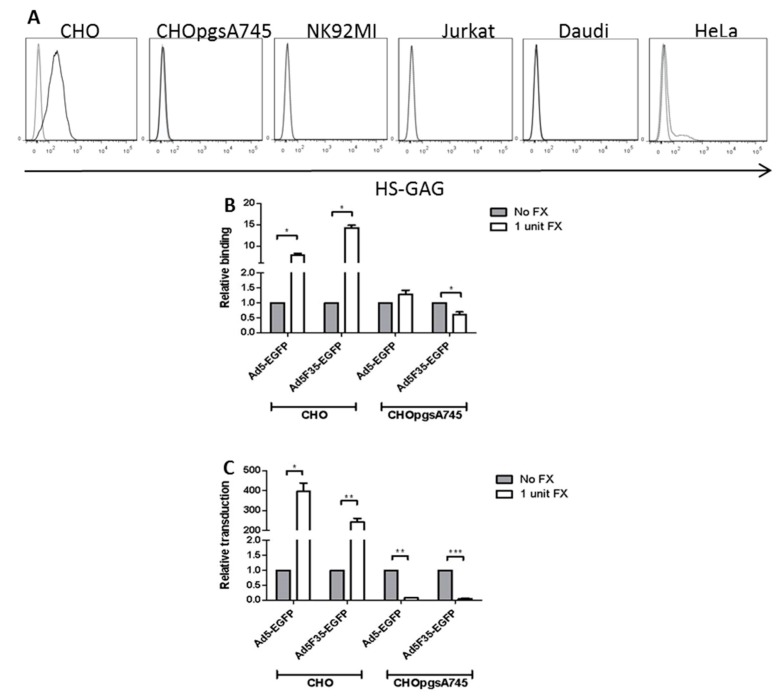
Heparan sulphate proteoglycan (HSPG) expression in lymphoid cells and the role of HSPGs in FX-mediated adenovirus entry. (**A**) The expression of HSPGs on HeLa epithelial and the lymphoid cell lines was determined, along with CHO and HS-GAG-negative CHOpgsA745 cells (as positive and negative controls respectively) by flow cytometry using a monoclonal antibody which recognises the 10E4 epitope present on heparan sulphate glycosaminoglycans, a component of HSPG [27]; (**B**) To quantify virus binding, CHO and CHOpgsA745 cells were incubated with either Alex488-labelled Ad5-EGFP or Ad5F35-EGFP at a ratio of 10^4^ vp:cell in the presence or absence of FX on ice for one hour. Unbound virus was removed by washing in PBS and the fluorescence measured by flow cytometry; (**C**) CHO and CHOpgsA745 cells were incubated with either Ad5-EGFP or Ad5F35-EGFP at a 10^4^ vp:cell ratio in the presence or absence of FX. EGFP expression was measured after 24 h by flow cytometry. The values given are the geometric mean of EGFP expression relative to the geometric mean in the absence of FX. * = *p* < 0.05, ** = *p* < 0.01 and *** = *p* < 0.001 based on a one-sample t test. In (**B**,**C**), three experimental replicates were performed (*n* = 3) with all samples assayed in triplicate.

As FX has been proposed to alter virus binding by connecting the virus to cell-surface HSPGs, expression of HSPG was studied in the lymphoid cell lines. As controls, HSPG-positive normal CHO cells and an HSPG-negative mutant CHOpgsA745 cell line (which is deficient in xylosyltransferase, an enzyme that catalyses an initial step in the glycosaminoglycan component of HSPG biosynthesis) [26], were used. Both CHO cell lines are negative for CAR and CD46. The presence of cell-surface HSPG was analyzed by flow cytometry using the mouse monoclonal antibody termed 10E4 which detects a heparan sulphate epitope of HSPG [27]. As expected, wild-type CHO cells bound the 10E4 antibody strongly and the HSPG-deficient mutant CHOpgsA745 cells were negative for 10E4 binding (Figure 4A). While HeLa epithelial cells were positive for 10E4 binding, the lymphoid cell lines did not exhibit detectable 10E4 binding (Figure 4A). Previous studies have shown that the NK and T cell populations of PBL were negative for cell-surface HSPG while B cells were positive [28].

CHO mutant cell lines offer the possibility of directly investigating the role of HSPG expression in Ad binding and transduction. CHO and CHOpgsA745 cells were incubated on ice with Alexa Fluor 488-labelled Ad5-EGFP or Ad5F35-EGFP in the presence or absence of FX. Binding of Ad5-EGFP and Ad5F35-EGFP virus particles to CHO cells was enhanced by FX by approx. 7- and 15-fold respectively. However, FX had a negligible effect on Ad5-EGFP binding but significantly reduced Ad5F35-EGFP binding to CHOpgsA745 cells (Figure 4B). Incubation of CHO and CHOpgsA745 cells with Ad5-EGFP or Ad5F35-EGFP at 37 °C revealed that FX significantly enhanced Ad5-EGFP and Ad5F35-EGFP transduction of CHO cells and dramatically reduced both Ad5-EGFP and Ad5F35-EGFP transduction of CHOpgsA745 cells (Figure 4C).

## 4. Discussion

Viruses have evolved many mechanisms to attack, subvert and control host cells. Initial interactions between viruses and host cells involve interactions at the cell surface followed by exploitation of host cell structures and pathways following internalization of the virus. Although intracellular structures and components have been a major focus of virology research, extracellular factors have relatively recently been identified as modulating agents of virus entry and evasion of the host humoral immune system. Factor X (FX) binds to the major adenovirus (Ad) capsid protein, the hexon, of most Ad types and has been proposed to both enhance Ad entry into cells by interaction with cell-surface heparan sulphate proteoglycans (HSPG) and protect the virus against inactivation by complement and IgM [12,13,14,15,29]. FX may therefore play a significant role in mediating Ad infection in vivo. Previous reports have mainly focused on the effects of FX on Ad binding, uptake and expression in epithelial cells, such as human HepG2, SKOV3 or A549 cells or nonhuman CHO cells [8,9,10,11,12,13,15,24]. In this study, interactions between Ads, FX and human lymphoid cell lines or primary lymphocytes were examined since it seems likely that Ads may encounter lymphoid cell types during natural infections [30] or when administered intravenously as gene therapy vectors [4,5,6,7]. Two Ads were chosen for virus binding and cell transduction studies that differed only in the origin of the viral fiber protein, namely Ad5-EGFP and Ad5F35-EGFP. In the latter virus, the Ad5 fiber was replaced with that of Ad35. This replacement alters the tropism of the virus from CAR-interacting (Ad5) to CD46-interacting (Ad5F35). Both viruses contained the same Ad5 hexon protein and an EGFP transgene for detection of virus-mediated transduction of target cells.

A panel of lymphoid cells was chosen to represent the main classes of blood lymphocytes: Jurkat cells (a T-cell line), Daudi cells (a B-cell line) and NK92MI (an NK-cell line). Jurkat and Daudi cells showed transduction by Ad5-EGFP and Ad35-EGFP, in agreement with previous studies [19]. NK92MI cells were transduced by Ad5F35-EGFP but not by Ad5-EGFP, also as described [17], consistent with the expression of CD46 but not CAR on the NK92MI cell surface (Figure 1).

FX significantly reduced the level of Ad5F35-EGFP transduction of all lymphoid cell lines as well as the T- and B-cell populations of peripheral blood lymphocytes. Binding of fluorescently-labelled Ad5F35-EGFP to the lymphoid cell lines was reduced by FX treatment, this varied between cell lines with Daudi and NK92MI being most affected. FX also reduced Ad5-EGFP transduction of Daudi and Jurkat cells but had no effect on fluorescently-labelled Ad5-EGFP binding to these cell lines, suggesting that FX may interfere with intracellular processes involved, for example, in trafficking Ad5 virus particles to the nucleus. In contrast, Ad5-EGFP binding to and transduction of HeLa epithelial cells was stimulated by FX, as shown in many other studies with a variety of epithelial cell lines [8,9,10,11,12,13,24]. However, transduction of HeLa cells by Ad5F35-EGFP was greatly reduced by FX, even though binding of this virus to HeLa cells was greatly enhanced by FX. These results point to cell-type differences in Ad binding to and transduction of susceptible human cells—overall, there was a stimulation of Ad transduction of epithelial cells but a reduction in transduction of lymphoid cells. Furthermore, the presence of the Ad35 fiber appears to confer different virus binding and cell transduction properties on Ad5F35-EGFP compared to Ad5-EGFP in response to FX, even in the same cell line.

The intracellular process that is modulated by FX during Ad5-EGFP and Ad5F35-EGFP transduction of lymphoid and HeLa cells remains to be determined. Previous studies on the effects of FX on Ad5F35 in CHO cells suggested that FX has increased stability in the lower pH environment associated with the late endosome and that Ad5F35 particles could be detected in late endosome/lysosomes [31]. Therefore it is possible that FX retains the Ad5F35-EGFP virus in late endosomes, resulting in reduced escape into the cell and hence reduced transduction. Previous studies on Ad5 infection of Daudi cells (in the absence of FX) have shown that Ad5 was detected in both late and early endosomes [19]. Information on intracellular trafficking of Ad5F35 in lymphoid cells in the presence of FX is not currently available, therefore this process needs to be studied to understand the reduction of transduction found in this study.

Models for FX-mediated entry of Ad5 into epithelial cells include its role in the formation of a bridge between hexon and the host cell. As HSPG has been shown to be dispensable in vivo for this process [14], it is essential to investigate the mechanism of FX action involved in modulation of attachment molecule-dependent entry. In this study, flow cytometric analysis of cell-surface HSPG revealed the lymphoid cell lines to be HSPG-deficient, in contrast to HeLa and normal CHO cells. While the reduction can be related in part to the absence of HSPG, other cellular mechanisms can also be involved. Of interest in this respect was the finding that, in an HSPG-deficient mutant CHO cell line (CHO-pgsA745), binding of and transduction by Ad5F35-EGFP was also reduced in the presence of FX. This suggests that, in addition to the lack of HSPGs, other FX-mediated mechanisms are involved. Since CHOpgsA745 cells lack CD46, and FX treatment reduced Ad5F35-EGFP binding and transduction, this might suggest that other, unidentified molecules are responsible for these downregulatory effects of FX on Ad5F35-EGFP binding to and transduction of CHO cells. Such mechanisms may operate at the cell surface and/or within the cell, since previous studies have suggested that post-internalization pathways may be affected by FX in normal CHO cells transduced by Ad5F35 viruses [15,29].

In conclusion, lymphoid cells in culture or in blood appear to be protected from Ad infection by either a low expression of CAR (which protects cells from infection by most Ads) or by a FX-mediated downregulation of binding and intracellular trafficking by Ads containing a fiber that binds CD46, a complement inhibitor molecule expressed on immune cell types.
